# Surgical Aortic Valve Replacement in Patients Aged 50 to 70 Years: Mechanical or Bioprosthetic Valve? A Systematic Review

**DOI:** 10.3390/healthcare11121771

**Published:** 2023-06-15

**Authors:** Evangelia Sigala, Martha Kelesi, Dimitrios Terentes-Printzios, Georgios Vasilopoulos, Theodoros Kapadohos, Dimitrios Papageorgiou, Alexia Tzatzou, Charalambos Vlachopoulos, Areti Stavropoulou

**Affiliations:** 1 Department of Nursing, University of West Attica, 12243 Athens, Greece; 2 1st Department of Cardiology, Hippokration Hospital of Athens, 11527 Athens, Greece; 3 Faculty of Health, Science, Social Care and Education, Kingston University, Kingston upon Thames KT2 7LB, UK

**Keywords:** aortic valve replacement, aortic valve prosthesis, complications, survival, long-term

## Abstract

Although transcatheter aortic valve implantation has emerged as a very attractive treatment option for severe aortic valve disease, surgical aortic valve replacement (SAVR) is still considered the standard-of-care, particularly in younger patients. However, selecting the appropriate type of valve prosthesis for this patient population can pose challenges. The aim of this systematic review was to investigate morbidity and mortality in patients aged 50–70 years who have undergone a first-time SAVR, and to define and compare the outcomes of mechanical valve (MV) and biological valve (BV) prosthesis. A systematic search was conducted to investigate the clinical outcomes of MVs and BVs in patients aged 50–70 years following the PRISMA guidelines. A total of 16,111 patients were included in the studies with an average follow-up of 10 years. A total of 16 studies were selected, 12 of which included propensity-score-matching (PMS) analysis and 4 of which obtained results via multivariate analysis. The vast majority (13 studies) showed no greater survival benefit in either MVs and BVs, while three studies showed an advantage of MVs over BVs. Regarding complications, bleeding was the most common adverse event in patients undergoing MV replacement, while for patients receiving BV prosthesis, it was structural valve deterioration and reoperation. Although the data suggest that the BV option could be a safe option in patients younger than 70 years, more studies with contemporary data are needed to draw firm conclusions on the risks and benefits of BV or MV in SAVR. Physicians should individualize the surgical plan based on patient characteristics.

## 1. Introduction

Severe aortic valve disease is a major contributor to cardiovascular morbidity and mortality worldwide [1]. Valvular diseases account for a significant proportion of cardiovascular disease cases, and the number of heart valve replacements performed globally was approximately 290,000 in 2003 [2]. This figure is expected to rise significantly, reach an estimated 850,000 in the coming decades [1,2]. Among these cases, AVD stands out as the most common, accounting for 44.3% of all instances [2]. The epidemiology of severe aortic valve disease highlights the importance of appropriate management of this condition to improve patient outcomes and reduce the burden of cardiovascular disease. Although transcatheter aortic valve replacement (TAVR) has gained popularity in recent years due to its less invasive nature, reduced hospitalization time, and comparable short-term surgical outcomes, surgical aortic valve replacement (SAVR) remains the gold-standard therapy for many patients, especially younger patients with a longer anticipated life expectancy [3,4,5]. Depending on the type of valve prosthesis, the choice between biological versus mechanical prosthesis in middle-aged patients has been controversial for decades [6]. In general, a bioprosthetic valve (BV) may be preferable for patients who prioritize avoiding the long-term anticoagulation therapy associated with an MV prosthesis. On the other hand, younger patients usually opt for MV prostheses due to its greater durability, especially when there is a reasonable life expectancy [3]. However, according to the 2020 ACC/AHA Guideline for the Management of Patients with Valvular Heart Disease, a BV may be considered for young patients who have a higher risk of bleeding and a higher risk of anticoagulation-related complications with an MV [7,8,9]. This suggests that a BV may be a viable option in selected patients younger than 70 years of age.

Although the ACC/AHA and ESC/EACTS guidelines consistently recommend MV prostheses in patients younger than 50 years of age, there is a growing trend towards considering BV prostheses for patients between 50 and 65 years of age, due to advancements in the field [3,10]. Recent studies have shown a 32.8% increase in the annual number of BV implantations in patients aged 18–50 years between 1997 and 2014 [7]. As the field continues to develop with new advancements in both MVs and BVs and ongoing research into non-vitamin K antagonist oral anticoagulants (NOACs) comparing the outcomes of survival and complications by valve type could provide valuable insights [9].

The aim of this systematic review was to assist in improving informed shared decision-making for patients by studying the long-term survival of patients aged 50–70 years who have undergone a single SAVR for the first time with or without concurrent coronary artery bypass grafting (CABG) surgery, while also defining the most commonly reported complications.

## 2. Method

A PubMed and Scopus search following the guidelines of PRISMA (Preferred Reporting Items for Systematic Reviews and Meta-Analyses, Figure S1 in Supplementary Materials) [11] was conducted to systematically identify studies that respect the following assumptions:

(1) To be included in the analysis, the studies were required to have made a direct comparison between MV and BV prostheses.

(2) The types of studies were propensity-score-matched cohorts (PMS), retrospective studies, or randomized controlled trials (RCTs). Review articles, case reports, and editorials were excluded.

(3) The minimum follow-up time, which consisted of survival data, was 5 years.

(4) All patients were older than 18, but most of them were around 50 years and younger than 70 years.

(5) All patients had a first-time isolated AVR or concomitant CABG and had not previously undergone any other type of cardiac surgery. Studies in patients with concomitant aortic root disease or other valve replacements were excluded.

The last search was conducted on 10 November 2022. Title, abstract screening, and full-text eligibility were assessed by 2 independent investigators (E.S. and A.T.). Any issues were resolved through consensus or discussion with an experienced colleague (A.S.). Pubmed and Scopus databases were searched for studies that compared the long-term survival and potential complications, such as re-operation rate, major bleeding, and stroke in patients under the age 70 years. A reference list review was also performed to identify articles missed by PubMed and Scopus searches. Combined keywords used for our search were: “mechanical”, “biological”, “bioprosthesis”, “bioprostheses”, “bioprosthetic”, “aortic”, and “AVR” or “SAVR”. We manually identified selected studies by examining their titles or using other available information to confirm that only patients aged between 50 and 70 years and with first-time single-valve SAVR were included.

## 3. Results

### 3.1. Selected Studies

The general study characteristics are presented in Table 1 and in Table S1 (Supplementary Materials). In total, 16 studies from 11 countries were included, 12 of which reported results from PMS cohorts [12,13,14,15,16,17,18,19,20,21,22,23], and a further 4 studies reported results from multivariate analysis [24,25,26,27]. Of those studies, only one was RCT study [26]. Survival outcomes are presented in Table 2 and complications in Table 3. Complications are shown in Table 4. The total number of patients analyzed was 16,111.

#### 3.1.1. Long-Term Survival

All studies provided data on 10-to-15-year survival (Table 1). Among these, 13 studies reported that long-term survival is comparable with BV and MV (Table 2 and Table 3), while 3 studies with propensity-matching found a significant mortality benefit of MVs over BVs (Table 2) [15,19,23].

Studies that support the use οf the MVs in patients between 50–70 years have shown similar results over a period of almost a decade. In the most recent study of Kyto et al. [15], which included 1152 patients aged 50–70 years, the 10-year mortality was lower in those who received MVs compared to BVs (18.6% vs. 27.6%; HR 0.72; CI 0.54–0.97, *p* = 0.028). Glazer et al. [19] also found a lower 15-year mortality in 2198 patients aged 50–69 years who received MVs (50% vs. 59% with BVs; *p* = 0.006) but no difference was observed in cardiovascular death. In their subgroup analysis, MVs aged 50–59 years were superior to BVs in terms of survival (*p* = 0.026), but this finding was not confirmed in those aged 60–69 years (*p* = 0.54). Similarly, in a study by Brown et al. [23] involving 440 patients aged 50–70 years, the 10-year unadjusted survival rate was higher with MVs compared to BVs (65% vs. 50%, *p* < 0.01).

On the other hand, the results of 13 studies, including the only contemporary prospective-matched cohort [27], demonstrate comprable long-term survival. By way of indication, we provide the results of three significant studies. Stassano et al. [26] conducted a study with 310 subjects with a mean age of 64 years and found no significant difference in 13-year survival between the two valve types (mortality rates: MVs 27.5% vs. BVs 30.6%, *p* = 0.06). Chiang et al. [21], in a PMS cohort (1001 pairs) of patients aged 50–69 years who received MVs or BVs, reported that no difference in 15-year survival was observed between the groups (MVs 62% vs. BVs 61%, *p* = 0.074). Rodriguez et al. [12] reported propensity-matched data on 5217 patients aged 50–65 years (PMS 2:1; 1822 MV vs. 911 BV) from a multicenter observational database (SPAVALVE study) and found no significant difference in long-term survival between the two groups (HR 1.14; CI 0.88–1.47; *p* = 0.33).

#### 3.1.2. Complications

According to the guidelines for reporting mortality and morbidity after cardiac valve intervention [28], bleeding, stroke, thrombosis, SDV, and reoperation were reported more frequently as serious adverse prosthesis-related events (MAPE). Bleeding was a more commonly observed complication in patients with MV prostheses across most studies. Additionally, as shown in Table 4, 111 studies reported that more frequent reoperation was required for patients with BVs. Finally, three studies showed no significant difference in terms of complications observed between the two groups [13,14,16]. The three studies which showed better survival outcomes for the MV group compared to BV group were for patients aged 50–70 years. Kyto et al. [15] found that patients who received a BV prosthesis had a greater risk of re-operation (BVs 8.5% vs. MVs 1.4%, *p* = 0.009). Similarly, Glazer et al. [19] (HR 2.36, CI 1.25–3.39, *p* = 0.001) and Brown et al. [23] (freedom of reoperation, MVs 98% vs. BVs 91%, *p* < 0.01) found that patients with an MV had a lower risk of reoperation. The same studies report more frequent hemorrhagic complications in the MV groups compared to the BV groups (Table 3).

#### 3.1.3. Quality of Life

In terms of quality of life (QOL), numerous studies have either reported comparable outcomes with mechanical and BVs, or the results have also been conflicting [8,13]. Stocco et al. [13], in addition to monitoring possible complications, also provided results on QOL. Although there was no significant difference when assessing the type of valve prosthesis, it was found that patients with a mechanical valve reported complaints about the sound of the valve (24%) and frequency of blood tests (37%), as well as fear of bleeding (21%). Patients with a BV expressed fear of prosthesis-related complications (41%) and reoperation (48%). This specific study is one of the few that has examined QOL concurrently with clinical outcome, and it is worth noting that no significant difference was found in terms of survival.

## 4. Discussion

The aim of this systematic review was to address whether there is a strong risk–benefit ratio for BV versus MV in middle-aged patients undergoing SAVR. Overall, the number of studies reporting differences in survival is very limited. Regarding complications, the reported findings appear more consistent with MV patiens having an increased risk for bleeding events, and BV patients having a higher risk of SVD and reoperation.

### 4.1. Survival

Studies comparing the long-term survival outcomes of middle-aged patients who undergo SAVR with MV or BV prostheses have been the subject of much research and debate. With improvements in medical technology and surgical techniques, patients and healthcare providers have more treatment options than ever before. However, choosing the right treatment option can be challenging, especially when considering the potential long-term impact on patient survival. Understanding the results of these studies may help guide treatment, shared decision making, and improve patient outcomes.

A large PMS study [19] involving 2198 patients aged 50–69 years found that the survival rate at 15 years was significantly higher in patients who received MV prosthesis (MV 59% vs. BV 50%, *p* = 0.006). However, in a sub-analysis of the sample, no significant difference was found between the 60–69 age group (*p* = 0.54). In practice, this means that the younger the patient, the higher the risk of death for those who decided to have BV. The study by Chiang et al. [21] contradicted these results and found that the survival rate of 2002 patients (PMS 1:1) aged 50–69 years was similar in sub-analyses. At this point it should be noted that both studies were based on propensity-matching scores, a type of data analysis that does not allow an exact cause of death to be determined, only the end-result. One common notion among all these studies is that none reported significanlty improved survival with biological over the mechanical prostheses in this age group. Further research is required due to improved technical and surgical skills, the newer generation of valves with lower INR goals, and the choice of TAVR over reoperation.

According to several studies, especially those involving younger patients (<65 years), a significant advantage of MVs over BVs has been found [15,19,23]. In a meta-analysis [29] that included five studies with patients aged 50–70 years (all included in our study), a statistically significant survival benefit with MVs was found; however, individually, four of the five studies reported no difference in survival. It is important to note that the studies included in this meta-analysis had a limited follow-up period (<10 years). Similarly, a more recent meta-analysis by Tasoudis et al. [30] showed that MVs were associated with higher long-term survival compared to BVs, particularly in patients aged between 50 and 70 years. The authors concluded that in younger patients, MVs may be the preferred choice due to their superior durability, but in older patients, BVs may be a reasonable option given their lower risk of thromboembolic events and need for long-term anticoagulation. The superior durability and the need for anticoagulation are related to the material used in valve construction. Pyrolytic carbon is typically used for MVs, while BVs are typically made using bovine, equine, or porcine pericardium or porcine aortic valves. Another factor that may contribute to better long-term survival is prosthesis mismatch, which is more common in patients with BVs [31].

Considering that the timing of redo surgeries occurs usually between 10 and 20 years, but also that at the time of the specific studies, there were no data on transcatheter valve-in-valve replacement beyond 5 years, we understand that objections could be raised to drawing a conclusion for the choice of BV at a young age [8]. It is also important to note that the results of meta-analyses should be interpreted with caution, as they are based on pooled data from multiple studies and may be affected by differences in study design and patient populations. Overall, while some studies suggest that MVs in certain patient populations, such as younger patients or patients at higher risk of reoperation, may be associated with better survival outcomes, the majority of studies do not indicate a significant difference in survival rates between the two prosthetic valve types. However, this should not be the sole consideration for clinicians when deciding between MVs or BVs, since survival outcomes can be influenced by age, frailty, bleeding risk, and other comorbidities [3,8]. Since there is no strong and consistent mortality benefit in this population, it is important that clinicians discuss the options with their patients and weigh the risks and the benefits of each type of prosthesis based on the individual patient-specific risk profile.

### 4.2. MAPE

After reviewing all selected studies, it can be concluded that, despite advancements in the medical management of aortic valve disease, patients undergoing SAVR are still susceptible to developing MAPE. Additionally, when comparing the complications of each valve prosthesis, it is difficult to determine which is more dangerous due to the different risks and benefits associated with each valve prosthesis and the individual circumstances of the patient. However, it has been consistently observed that patients with MV are at greater risk of major bleeding due to the need for lifelong anticoagulant therapy. On the other hand, BVs typically have a shorter lifespan and may require future reoperation.

Careful consideration of complications depending on the choice of valve type could provide a more accurate estimate as there was no significant advantage in survival over a particular valve type according to studies. It is worth mentioning that a large Spanish multicenter observational study (SPAVALVE, 2:1 PMS, 1822 MV and 911 BV, 15-year survival outcome) [12] in patients aged 50–65 years confirms our overall view of complications according to MAPE. A higher rate of major bleeding events was reported in the MV group (*p* = 0.004) and the need for reoperation was significantly higher in the BV group (*p* < 0.001). However, the investigators emphasized that the outcomes for procedures performed after 2009 have changed, with a significant reduction in stroke, major bleeding and reintervention related to AVR (all *p* < 0.001). The incorporation of newer technical aspects such as calcification-resistant properties have improved the durability of newer BV prostheses.

Although the SPAVALVE study suggests that younger patients have comparable survival outcomes with BVs, it shoud be emphasized that the risk of reoperation in the age group <70 years is associated with a higher risk of mortality [12,31]. In addition, a follow-up period of up to 15 years has been established in previous studies. As life expectancy in developed countries has increased to over 80 years, we need longer follow-up periods to better estimate the risk–benefit ratio in this age group.

It was emphasized that patient preferences are of high importance in treatment planning and therefore, the American Heart Association revised their guidelines in 2017: for patients between 50–70 years, the choice of prosthesis can be individualized and for some, the decision of redo-TAVI after thorough screening could be possible in the future [10]. The possibility of redo-TAVI is a logical argument and, as mentioned, a safer option unless the valve size is 21 mm or smaller than the initial surgery. In addition to the small aortic annulus, in the case of such factors as, age < 60, preoperative lifelong OAC, metabolic syndrome, and hyperparathyroidism, an MV is preferable [3,5,10]. In the case of such factors as age > 70; age < 60 but with a life expectancy of < 10 years; age < 60 but desire to conceive, hazardous occupation, or pregnancy; reoperations for valve thrombosis, high risk of bleeding or contra-indication for OAC; and end-stage renal failure, a BV is preferable [3,8,10].

Based on these facts, when advising patients, we should keep in mind that the options that appear to offer short-term safety may not objectively assess the possible long-term risk [8]. Again, based on the MAPE results of the selected studies, we conclude that the choice of valve prosthesis is a personal decision that should be made in consultation with a health-care provider, taking into account the patient’s individual medical history and preferences.

As final point, we mentioned that Stocco et al. [13] provided results on QOL. The holistic approach seems extremely useful for drawing safe conclusions. A subjective evaluation combined with survival results of possible complications is the combination that should be present in future studies to further support the rational arguments for the choice of the prosthesis. It is important that patients fully understand the risks and benefits of each type of valve prosthesis, as well as the potential impact on their quality of life. With the right education and support, patients can make an informed decision that meets their individual needs and preferences.

Based on this, when advising patients, we should take into account each individual patient’s personal preferences, medical history, and try to estimate the long-term risks of each option and share these details with the patient, as ultimately, the choice of valve prosthesis is a shared decision.

#### Clinical Implications: Innovations and Alternatives

Prosthetic heart valve technology is advancing, with research focused on improving safety, durabiltiy, and effectiveness. TAVI may be a better option than SAVR for middle-aged patients if they meet certain criteria [5] and valve-in-valve (ViV) reoperation may be an alternative for young patients choosing BV, though the efficacy of ViV for patients with smaller size of bioprostheses in the initial operation (21 mm or smaller) remains uncertain. Based on currently availably data, there appears to be insufficient evidence to support lowering the age limit for BV implantation below 60 years to improve long-term survival.

Ongoing research in the field of valve prostheses is expected to bring significant developments that could have a major impact on the treatment of AVS in younger patients. One promising area of research is the development of MVs that require lower anticoagulant target INR levels, as current guidelines do not recommend the use of NOACs in patients with MVs [3,9,32,33]. Another potential area of advancement is improved durability [34,35]. However, there are still many challenges to be overcome before tissue-engineered heart valves can be widely used in clinical practice, and in the meantime, traditional aortic valve prostheses remain an important treatment option.

Overall, we expect continuous advancements in the field of prosthetic valves and SAVR that aim to improve outcomes, reduce complications, and provide patients with a more personalized treatment option. Until then, it is important for the patients to have a clear understanding of the pros and cons of each option so they can make an informed decision that aligns with their personal preferences.

## 5. Limitations

When reviewing this topic, there are certain limitations that need to be considered. One major limitation is the scarcity of RCTs that would provide high-quality data on this topic. Another limitation of the selected studies was the different uses of terms to indicate survival (or mortality) and complications outcomes, and the fact that the age of the participants in most studies was highly variable. To be able to draw reliable conclusions about the optimal choice of valve type, further studies in this age group are needed.

Additionally, there may be variations in surgical techniques and postoperative management that could impact outcomes and make direct comparisons between studies difficult. It is also important to consider individual patient factors, such as comorbidities and lifestyle, when making decisions about valve selection, which may not be fully captured in the available literature. Finally, advancements in technology and techniques may result in changes to current recommendations and outcomes in the future. Due to tremendous progress in the past decade due to TAVR, more research on ViV TAVR is needed to gain a better understanding on this field.

## 6. Conclusions

The main results of our review show that there was no significant advantage when it comes to survival, according to valve type prosthesis in patients aged 50–70 years who underwent SAVR for the first time. However, in terms of adverse events, it was consistently observed that patients who opted for a BV were at higher risk of reoperation, while those who chose an MV were more likely to experience major bleeding. Based on the currently available evidence, the selection of a prosthetic valve should be tailored to the individual risk-profile and life expectancy of the patient, while bearing in mind that the choice of valve also depends on the patient’s personal preferences.

## Figures and Tables

**Table 1:** General characteristics of included studies.

Studies included	16	
Number of countries	11	
Number of participants	16.111	
Main inclusion criterion	Isolated AVR or AVR/CABG *^1^	
Age range (years, min/max)	50–70	
Design of included studies	Retrospective	15
	Prospective	1
Method of data analysis	Propensity matching	12
	Multivariate analysis	4
Follow-up period (years)	10-year	5
	13-year	1
	15-year	10
Patient inclusion period (min/max)	1982–2019	

*^1^ Isolated aortic valve replacement; or AVR in combination with coronary artery bypass graft surgery (CABG).

**Table 2:** Long-Term survival from observational studies using propensity-score-matching analysis to compare mechanical versus bioprosthetic aortic valve replacement. * Concerns the number of the study in the reference list. Similarly, this applies to all the tables of the text.

Author, Year	Population (MV; BV)	Age (Years)	Mean Follow-Up (MV; BV, Years)	Outcome	Survival Rates (MV; BV), HR Associate with MVs	Conclusion
Rodriguez 2023 [12] *	2733 (1822:911, 2:1)	50–65	8.1 ± 4.8; 7.3 ± 4.8 (*p* < 0.001)	15-year survival	HR 1.14, 95% CI 0.88–1.46, *p* = 0.33	No significant difference.
Stocco 2021 [13]	116 (58:58, 1:1)	<65	Overall 8.5 ± 3.5 (no comparison)	10-year survival	9.2% vs. 83.4%, *p* = 0.09	Trend toward significance.
Vitanova 2021 [14]	428 (214:214, 1:1)	<60	Overall 7.6 ± 3.9 (no comparison)	10-year survival	<60 years: 89 ± 3.4% vs. 97 ± 1.9%, *p* = 0.06 >60 years: 79.1 ± 5.8% vs. 69.8 ± 4.4%, *p* = 0.83	<60 years: Trend toward significance.
Kyto 2019 [15]	1152 (576:576, 1:1)	50–70	overall 6.7 ± 2.6 (*p* = 0.169)	10-year all-caused mortality	all-caused mortality 18.6% vs. 27.6%, HR 0.72, CI 0.54–0.97, *p* = 0.02	Higher survival with MV.
Rodriguez 2018 [16]	166 (83:83, 1:1)	50–65	9.7 ± 4.3 & 17 years; 6.1 ± 3.1 snf 17 years (*p* = 0.001)	15-year survival	65%, HR 0.87, 95% CI 0.41–1.82, *p* = 0.71	No significant difference.
Alex 2017 [17]	236 (118:118, 1:1)	55–65	overall, 6.9 (no *p*-value)	15-year survival	60.6% vs. 46.4%, HR 1.16, 95% CI 0.69–1.94, *p* = 0.58	No significant difference.
Sakamoto 2016 [18]	56 (28:28, 1:1)	60–70	7.0 vs. 7.8 (*p* = 0.60)	15-year survival	88% vs. 85%, *p* = 0.73	No significant difference.
Glazer 2016 [19]	2198 (1099:1099, 1:1)	50–69	6.7 vs. 6.6 years (no *p*-value)	15-year survival	59% vs. 50% HR = 0.75, 95% CI 0.60–0.92, *p* = 0.006	Higher survival with MV.
Roumieh 2015 [20]	120 (60:60)	55–65	10.7 vs. 8.8 (no *p*-value)	15-year survival	53% vs. 54%, *p* = 0.95	No significant difference.
Chiang 2014 [21]	2002 (1001:1001, 1:1)	50–69	10.9–10.6 (no *p*-value)	15-year survival	62% vs. 61%, *p* = 0.74	No significant difference.
McClure 2014 [22]	722 (361:361, 1:1)	<65	6.7 vs. 7 (no *p*-value)	15-year survival	75% vs. 65%, *p* = 0.75	No significant difference.
Brown 2008 [23]	440 (220:220, 1:1)	50–70	9.1 vs.6.1 (no *p*-value)	10-year survival	68% vs. 50%, HR = 0.48, 95% CI 0.35–0.67, *p* < 0.01	Higher survival with MV.

**Table 3:** Long-term survival from observational studies performing multivariable analysis to compare mechanical versus bioprosthetic aortic valve replacement.

**Author, Year**	**Population (MV; BV)**	**Age (Years)**	**Mean Follow-Up (MV; BV, Years)**	**Outcome**	**Survival Rates (MV; BV), HR Associate with MVs**	**Conclusion**
Malvindi 2021 [24]	977 (359:618)	50–69	9.8 vs. 5.2 (*p* < 0.01)	15-year survival	50–59-year-old group: BV, HR 1.464, CI 0.788–2.724, *p* = 0.23 60–69-year-old group: BV, HR 1.117, CI 0.809–1.721, *p* = 0.39	No significant difference.
van Geldrop 2009 [25]	3924	50–70	8.5 vs. 6.1 (no comparison)	15-year survival	12.2 vs. 11.9 (simulated life expectancy, 40-year-old man)	No significant difference.
Stassano 2009 [26]	310 (155:155)	55–70	106 ± 28 months (no comparison)	13-year survival	Mortality: 41(27.5%) vs. 45 (30.6%), *p* = 0.60	No significant difference.
Carrier 2001 [27]	521 (363:158)	55–65	4 vs. 7 (*p* = 0.001)	10-year survival	66 ± 6% vs. 75 ± 4%, *p* = 0.20	No significant difference.

**Table 4:** Complications reported from observational studies using propensity score matching or performing multivariable analysis comparing mechanical versus bioprosthetic aortic valve replacement.

Author, Year	Population (MV; BV)	Age (Years)	Observation Period (Years)	Complications (BV; MV)	Conclusion
Rodriguez 2023 [12]	2733 (1822:911, 2:1)	50–65	15 years	Mechanical Valve: Major bleeding events, HR 0.65, 95% CI 0.49–0.87, *p* = 0.004 Bioprosthetic Valve: Reoperation, HR 3.04, 95% CI 1.80–5.14, *p* < 0.001	Bleeding risk: MV worse than BV Reoperation risk: BV worse than MV
Stocco 2021 [13]	116 (58:58, 1:1)	<65	10 years	Mechanical Valve: Major bleeding (3% vs. 20%, *p* = 0.09) Bioprosthetic Valve: Reoperation (8% vs. 0%, *p* = 0.9)	No significant difference.
Vitanova 2021 [14]	428 (214:214, 1:1)	<60, >60	10 years	Mechanical Valve: >60: Tendency in higher MACCE rates at 10 years (4.3 ± 3.1% vs. 9.1 ± 3.1%, *p* = 0.86) Bioprosthetic Valve: <60: Tendency in higher MACCE rates at 10 years (7.3 ± 5.3% vs. 4.6 ± 2.2%, *p* = 0.83)	No significant difference.
Kyto 2019 [15]	1152 (576:576, 1:1)	50–70	10 years	Mechanical Valve: Major Bleeding (16.9% vs. 21.5%, *p* = 0.40) Stroke (9.3% vs. 12.7%, *p* = 0.31) Bioprosthetic Valve: Endocarditis (7.3% vs. 3.7%, *p* = 0.01) Reoperation (8.5% vs. 1.4%, *p* = 0.009)	BV: Higher risk of reoperation and infective endocarditis.
Rodriguez 2018 [16]	166 (83:83, 1:1)	50–65	15 years	Mechanical Valve: More major adverse cardiovascular complications (30% vs. 15%, *p* = 0.07) Major bleedings (15% vs. 6.3%, *p* = 0.06) Stroke (11% vs. 7.6%, *p* = 0.44) Cardiac related rehospitalization (33.7% vs. 21.5%, *p* = 0.06) Bioprosthetic Valve: Reoperation (2.5% vs. 6.3%, *p* = NS)	No significant difference.
Alex 2017 [17]	236 (118:118, 1:1)	55–65	15 years	Mechanical Valve: Cumulative incidence of MAPE (major adverse prosthesis-related event) 53.3% vs. 24.5%, HR 0.65, 95% CI 0.37–1.14, *p* < 0.12 Bioprosthetic Valve: Reoperation (26.0% vs. 5.4%), HR 0.24, 95% CI 0.09–0.68 *p* < 0.01.	BV: Higher risk of reoperation.
Sakamoto 2016 [18]	56 (28:28, 1:1)	60–70	15 years	Mechanical Valve: Thromboembolism (0.58% vs. 0.35% patient per year, *p* < 0.001) Hemorrhage (0.34% vs. 0.12% patients per year, *p* < 0.001)	MV: Higher risk of thromboembolism and hemorrhage.
Glazer 2016 [19]	2198 (1099:1099, 1:1)	50–69	15 years	Mechanical Valve: Risk for major bleeding, HR 0.49, 95% CI 0.34–0.70, *p* < 0.001 Bioprosthetic Valve: Risk for aortic valve reoperation, HR 2.36, 95% CI 1.42–3.94, *p* = 0.001	MV: Higher bleeding risk BV: Higher risk of reoperation.
Roumieh 2015 [20]	120 (60:60, 1:1)	55–65	15 years	Mechanical Valve: Freedom from structural valve deterioration (64 ± 12 vs. 93 ± 5%, *p* = 0.003) Freedom from redo AVR (73 ± 11 vs. 91 ± 5%, *p* = 0.04) Freedom of Endocarditis (83 ± 8 vs. 98 ± 2%, *p* = 0.05) Freedom of Cerebrovascular events (83 ± 8 vs. 97 ± 3%, *p* = 0.03) Bioprosthetic Valve: Freedom of bleeding events (88 ± 6 vs. 77 ± 10%, *p* = 0.03)	MV: Higher bleeding risk. BV: Higher risk of structural valve deterioration and redo AVR, infective endocarditis, cerebrovascular events.
Chiang 2014 [21]	2002 (1001:1001, 1:1)	50–69	15 years	Mechanical Valve: Stroke (7.7% vs. 8.6%), HR 1.04, 95% CI 0.75–1.43) Bleeding (13% vs. 6.6%), HR 1.75, 95% CI 1.27–2.43) Bioprosthetic Valve: Reoperation (12.1% vs. 6.9%), HR 0.52, 95% CI 0.36–0.75)	MV: Higher Stroke and bleeding risk BV: Higher reoperation risk.
McClure 2014 [22]	722 (361:361, 1:1)	<65	15 years	Mechanical Valve: Freedom from reoperation at 18 years (55% vs. 95%, *p* = 0.002) Bioprosthetic Valve: Freedom of major bleeding event at 18 years (98% vs. 78%, *p* = 0.002)	MV: Higher bleeding risk. BV: Higher reoperation risk.
Brown 2008 [23]	440 (220:220, 1:1)	50–70	10 years	Mechanical Valve: Hemorrhagic complication necessitating hospitalisation (15% vs. 7%, *p* = 0.01) Bioprosthetic Valve: Freedom of 10-year reoperation (91% vs. 98%, *p* < 0.01)	MV: Higher bleeding risk. BV: Higher reoperation risk.
Malvindi 2021 [24]	977 (359:618)	50–69 (2 groups)	15 years	Mechanical Valve: 60–69-year group Hemorrhagic complications (6.9% vs. 16.2%, *p* = 0.001) Bioprosthetic Valve: 50–59-year group Reintervention (26.3% vs. 2.6%; *p* < 0.001) 60–69-year group: Reintervention for valve dysfunction (20.9% vs. 4.8%; *p* = 0.02).	MV: Higher bleeding risk in the 60–69-year group. BV: Higher reoperation risk in the 50–59-year group. Higher reintervention for valve dysfunction in the 60–69-year group.
van Geldorp 2009 [25]	3934 (1074:2860)	50–70	15 years	Mechanical Valve: Bleeding risk (12% vs. 41%) Bioprosthetic Valve: Reoperation (25% vs. 3%)	MV: Higher bleeding risk. BV: Higher reoperation risk.
Stassano 2009 [26]	310 (155:155)	55–70	13 years	Bioprosthetic Valve: Valve failure and reoperation (*p* < 0.001, *p* = 0.003)	BV: Higher reoperation risk.
Carrier 2001 [27]	521 (363:158)	55–65	10 years (Multivariable analysis)	Bioprosthetic Valve: 10-year freedom rate from all valve-related complications: BV 83% ± 4% vs. MV 90% ± 7%, *p* = 0.01	BV: Higher risk of all valve-related complications.

## Data Availability

No new data were created or analyzed in this study. Data sharing is not applicable to this article.

## Supplementary Materials

The following supporting information can be downloaded at: https://www.mdpi.com/article/10.3390/healthcare11121771/s1, Figure S1: PRISMA 2020 flow diagram for new systematic review; Table S1: General characteristics of the selected studies.

## References

[1] Yadgir S., Johnson C.O., Aboyans V., Adebayo O.M., Adedoyin R.A., Afarideh M., Alahdab F., Alashi A., Alipour V., Arabloo J. (2020). Global Burden of Disease Study 2017 Nonrheumatic Valve Disease Collaborators (2020). Global, Regional, and National Burden of Calcific Aortic Valve and Degenerative Mitral Valve Diseases, 1990–2017. Circulation.

[2] Musumeci L., Jacques N., Hego A., Nchimi A., Lancellotti P., Oury C. (2018). Prosthetic Aortic Valves: Challenges and Solutions. Front. Cardiovasc. Med..

[3] Vahanian A., Beyersdorf F., Praz F., Milojevic M., Baldus S., Bauersachs J., Capodanno D., Conradi L., De Boinis M., De Paulis R. (2021). 2021 ESC/EACTS Guidelines for the management of valvular heart disease: Developed by the Task Force for the management of valvular heart disease of the European Society of Cardiology (ESC) and the European Association for Cardio-Thoracic Surgery (EACTS). Eur. J. Cardio-Thorac. Surg..

[4] Reineke D., Gisler F., Englberger L., Carrel T. (2016). Mechanical versus biological aortic valve replacement strategies. Expert Rev. Cardiovasc. Ther..

[5] Dahiya G., Kyvernitakis A., Joshi A.A., Lasorda D.M., Bailey S.H., Raina A., Biederman R.W.W., Kanwar M.K. (2021). Impact of transcatheter aortic valve replacement on left ventricular hypertrophy, diastolic dysfunction and quality of life in patients with preserved left ventricular function. Int. J. Cardiovasc. Imaging.

[6] Schnittman S.R., Adams D.H., Itagaki S., Toyoda N., Egorova N., Chikwe J. (2018). Bioprosthetic aortic valve replacement: Revisiting prosthesis choice in patients younger than 50 years old. J. Thorac. Cardiovasc. Surg..

[7] Korteland N.M., Top D., Borsboom G.J., Roos-Hesselink J.W., Bogers A.J., Takkenberg J.J. (2016). Quality of life and prosthetic aortic valve selection in non-elderly adult patients. Interact. Cardiovasc. Thorac. Surg..

[8] Head S.J., Çelik M., Kappetein A.P. (2017). Mechanical versus bioprosthetic aortic valve replacement. Eur. Heart J..

[9] Otto C.M., Nishimura R.A., Bonow R.O., Carabello B.A., Erwin J.P., Gentile F., Jneid H., Krieger E.V., Mack M., McLeod C. (2021). 2020 ACC/AHA guideline for the management of patients with valvular heart disease: Executive summary: A report of the American College of Cardiology/American Heart Association Joint Committee on Clinical Practice Guidelines. J. Am. Coll. Cardiol..

[10] Page M.J., Moher D., Bossuyt P.M., Boutron I., Hoffmann T.C., Mulrow C.D., Shamseer L., Tetzlaff J.M., Akl E.A., Brennan S.E. (2021). PRISMA 2020 explanation and elaboration: Updated guidance and exemplars for reporting systematic reviews. BMJ.

[11] (2022). Preferred Reporting Items for Systematic Reviews and Meta-Analyses. https://www.prisma-statement.org/?AspxAutoDetectCookieSupport=1.

[12] Rodríguez-Caulo E.A., Blanco-Herrera O.R., Berastegui E., Arias-Dachary J., Souaf-Khalafi S., Parody-Cuerda G., Laguna G., SPAVALVE Study Group (2023). Biological versus mechanical prostheses for aortic valve replacement. J. Thorac. Cardiovasc. Surg..

[13] Stocco F., Fabozzo A., Bagozzi L., Cavalli C., Tarzia V., D’Onofrio A., Lorenzoni G., Chiminazzo V., Gregori D., Gerosa G. (2021). Biological versus mechanical aortic valve replacement in non-elderly patients: A single-centre analysis of clinical outcomes and quality of life. Interact. Cardiovasc. Thorac. Surg..

[14] Vitanova K., Wirth F., Boehm J., Burri M., Lange R., Krane M. (2021). Surgical Aortic Valve Replacement—Age-Dependent Choice of Prosthesis Type. J. Clin. Med..

[15] Kytö V., Sipilä J., Ahtela E., Rautava P., Gunn J. (2020). Mechanical versus biologic prostheses for surgical aortic valve replacement in patients aged 50 to 70. Ann. Thorac. Surg..

[16] Rodríguez-Caulo E.A., Otero-Forero J.J., Sánchez-Espín G., Mataró M.J., Guzón A., Porras C., Villaescusa J., Such M., Melero J.M. (2018). 15 years outcomes following bioprosthetic versus mechanical aortic valve replacement in patients aged 50–65 years with isolated aortic stenosis. Cir. Cardiovasc..

[17] Alex S., Hiebert B., Arora R., Menkis A., Shah P. (2018). Survival and long-term outcomes of aortic valve replacement in patients aged 55 to 65 years. Thorac. Cardiovasc. Surg..

[18] Sakamoto Y., Yoshitake M., Matsumura Y., Naruse H., Bando K., Hashimoto K. (2016). Choice of aortic valve prosthesis in a rapidly aging and long-living society. Ann. Thorac. Cardiovasc. Surg..

[19] Glaser N., Jackson V., Holzmann M.J., Franco-Cereceda A., Sartipy U. (2016). Aortic valve replacement with mechanical vs. biological prostheses in patients aged 50–69 years. Eur. Heart J..

[20] Roumieh M., Ius F., Tudorache I., Ismail I., Fleissner F., Haverich A., Cebotari S. (2015). Comparison between biological and mechanical aortic valve prostheses in middle-aged patients matched through propensity score analysis: Long-term results. Eur. J. Cardio-Thorac. Surg..

[21] Chiang Y.P., Chikwe J., Moskowitz A.J., Itagaki S., Adams D.H., Egorova N.N. (2014). Survival and long-term outcomes following bioprosthetic vs mechanical aortic valve replacement in patients aged 50 to 69 years. JAMA.

[22] McClure R.S., McGurk S., Cevasco M., Maloney A., Gosev I., Wiegerinck E.M., Salvio G., Tokmaji G., Borstlap W., Nauta F. (2014). Late outcomes comparison of nonelderly patients with stented bioprosthetic and mechanical valves in the aortic position: A propensity-matched analysis. J. Thorac. Cardiovasc. Surg..

[23] Brown M.L., Schaff H.V., Lahr B.D., Mullany C.J., Sundt T.M., Dearani J.A., McGregor C.G., Orszulak T.A. (2008). Aortic valve replacement in patients aged 50 to 70 years: Improved outcome with mechanical versus biologic prostheses. J. Thorac. Cardiovasc. Surg..

[24] Malvindi P.G., Luthra S., Olevano C., Salem H., Kowalewski M., Ohri S. (2021). Aortic valve replacement with biological prosthesis in patients aged 50–69 years. Eur. J. Cardio-Thorac. Surg..

[25] Van Geldorp M.W., Jamieson W.E., Kappetein A.P., Ye J., Fradet G.J., Eijkemans M.J., Grunkemeier G.L., Bogers A.J., Takkenberg J.J. (2009). Patient outcome after aortic valve replacement with a mechanical or biological prosthesis: Weighing lifetime anticoagulant-related event risk against reoperation risk. J. Thorac. Cardiovasc. Surg..

[26] Stassano P., Di Tommaso L., Monaco M., Iorio F., Pepino P., Spampinato N., Vosa C. (2009). Aortic valve replacement: A prospective randomized evaluation of mechanical versus biological valves in patients ages 55 to 70 years. J. Am. Coll. Cardiol..

[27] Carrier M., Pellerin M., Perrault L.P., Pagé P., Hébert Y., Cartier R., Dyrda I., Pelletier L.C. (2001). Aortic valve replacement with mechanical and biologic prostheses in middle-aged patients. Ann. Thorac. Surg..

[28] Akins C.W., Miller D.C., Turina M.I., Kouchoukos N.T., Blackstone E.H., Grunkemeier G.L., Takkenberg J., David T.E., Butchart E.G., Adams D.H. (2008). Guidelines for reporting mortality and morbidity after cardiac valve interventions. Eur. J. Cardio-Thorac. Surg..

[29] Diaz R., Hernandez-Vaquero D., Alvarez-Cabo R., Avanzas P., Silva J., Moris C., Pascual I. (2019). Long-term outcomes of mechanical versus biological aortic valve prosthesis: Systematic review and meta-analysis. J. Thorac. Cardiovasc. Surg..

[30] Tasoudis P.T., Varvoglis D.N., Vitkos E., Mylonas K.S., Sá M.P., Ikonomidis J.S., Caranasos T.G., Athanasiou T. (2022). Mechanical versus bioprosthetic valve for aortic valve replacement: Systematic review and meta-analysis of reconstructed individual participant data. Eur. J. Cardio-Thorac. Surg..

[31] Pibarot P., Dumesnil J.G. (2009). Prosthetic heart valves: Selection of the optimal prosthesis and long-term management. Circulation.

[32] Gerdisch M.W., Sathyamoorthy M., Michelena H.I. (2022). The role of mechanical valves in the aortic position in the era of bioprostheses and TAVR: Evidence-based appraisal and focus on the On-X valve. Prog. Cardiovasc. Dis..

[33] Cheung D.Y., Duan B., Butcher J.T. (2015). Current progress in tissue engineering of heart valves: Multiscale problems, multiscale solutions. Expert Opin. Biol. Ther..

[34] Fioretta E.S., Motta S.E., Lintas V., Loerakker S., Parker K.K., Baaijens F.P., Falk V., Hoerstrup S.P., Emmert M.Y. (2021). Next-generation tissue-engineered heart valves with repair, remodelling and regeneration capacity. Nat. Rev. Cardiol..

[35] Fioretta E.S., Von Boehmer L., Motta S.E., Lintas V., Hoerstrup S.P., Emmert M.Y. (2019). Cardiovascular tissue engineering: From basic science to clinical application. Exp. Gerontol..

