# Supplementary material for: Surgical Aortic Valve Replacement in Patients Aged 50 to 70 Years: Mechanical or Bioprosthetic Valve? A Systematic Review
Source: Healthcare (Basel). 2023 Jun 15;11(12):1771. doi: 10.3390/healthcare11121771 (PMC10298478; doi:10.3390/healthcare11121771)
Supplement: Supplementary file 1 [file healthcare-11-01771-s001.zip › healthcare-2337762-supplementary.pdf]

Supplementary Materials

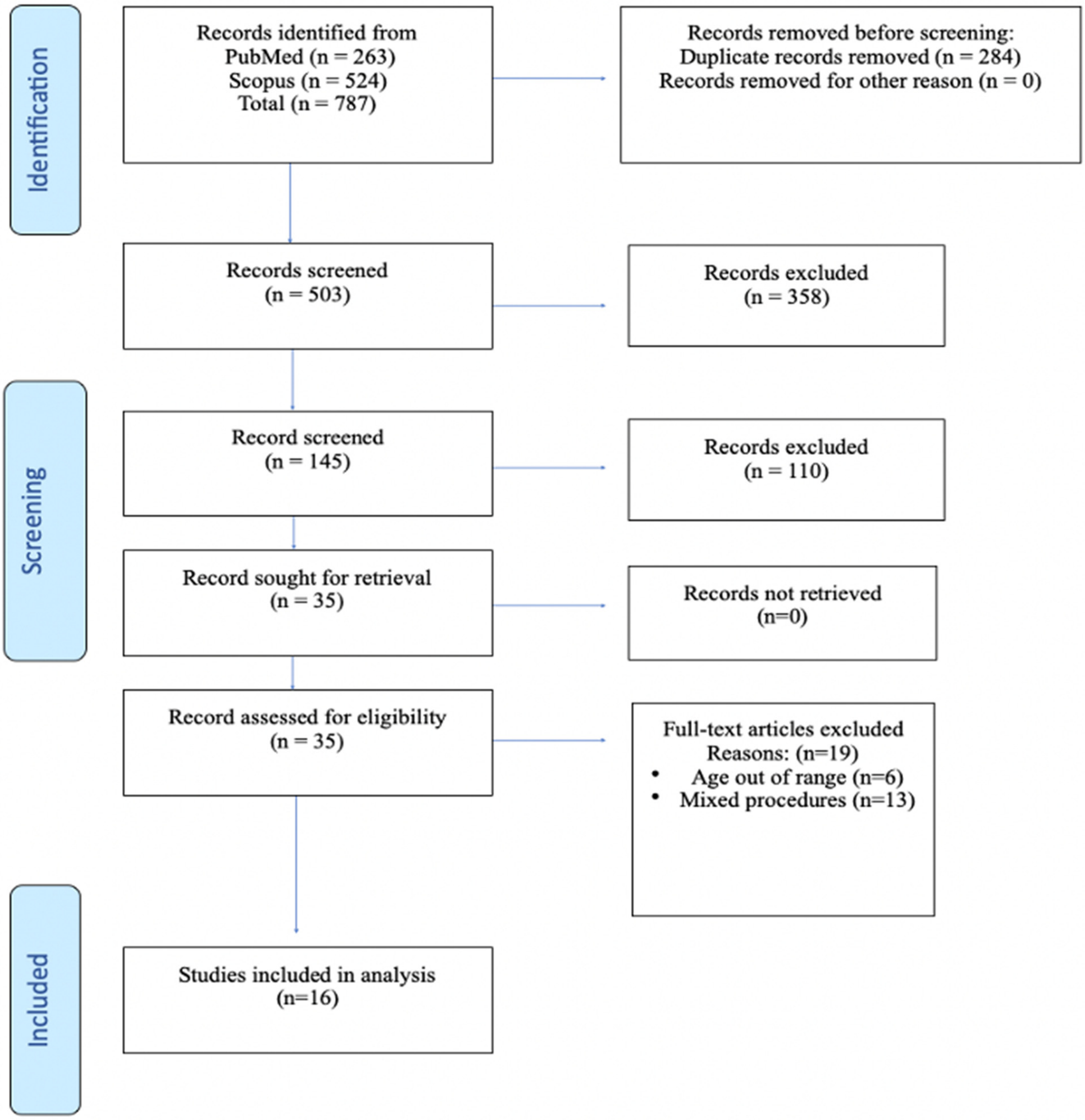

Figure S1. PRISMA 2020 flow diagram for new systematic review.

Table S1. General characteristics of the selected studies.

Author, Year	Country	Design	Patient Inclusion Period
Studies with propensity matching			
Rodriguez 2023 [12]	Spain	Retrospective	2000-2018
Stocco 2021 [13]	Italy	Retrospective	2002-2013
Vitanova 2021 [14]	Germany	Retrospective	2001-2015
Kyto 2019 [15]	Finland	Retrospective	2004-2014
Rodriguez 2018 [16]	Spain	Retrospective	2000-2015
Alex 2017 [17]	Canada	Retrospective	1995-2014
Sakamoto 2016 [18]	Japan	Retrospective	1995-2014
Glazer 2016 [19]	Sweden	Retrospective	1997-2013
Roumieh 2015 [20]	Germany	Retrospective	1996-2008
Chiang 2014 [21]	United States of America	Retrospective	1997-2004
McClure 2014 [22]	Boston	Retrospective	1992-2011
Brown 2008 [23]	United States of America	Retrospective	1991-2000
Studies with multivariate analysis			
Malvindi 2021 [24]	United Kingdom	Retrospective	2000-2019
Geldorp 2009 [25]	Canada	Retrospective	1982-2003
Stassano 2009 [26]	Italy	Retrospective	1995-2003
Carrier 2001 [27]	Canada	Prospective	1982-1999
